# Human Papillomavirus and Coronary Artery Disease in Climacteric Women: Is There an Association?

**DOI:** 10.1155/2019/1872536

**Published:** 2019-06-20

**Authors:** Luciane Maria Oliveira Brito, Haissa Oliveira Brito, Rita da Graça Carvalhal Frazão Corrêa, Clariano Pires de Oliveira Neto, Joyce Pinheiro Leal Costa, Sally Cristina Moutinho Monteiro, Flávia Castello Branco Vidal, Maria do Desterro Soares Brandão Nascimento, José Albuquerque de Figueiredo Neto, Rui Miguel Gil da Costa, Leonardo Victor Galvão-Moreira, Ismael Dale Cotrim Guerreiro da Silva

**Affiliations:** ^1^Tumor and DNA Biobank of Maranhão, Federal University of Maranhão, São Luís, Brazil; ^2^Molecular Oncology and Viral Pathology Group, Portuguese Institute of Oncology, Porto, Portugal; ^3^Federal University of São Paulo, São Paulo, Brazil

## Abstract

**Background:**

Cardiovascular diseases are leading causes of death worldwide. Recent studies suggest that infection by some viruses, including the human papillomavirus (HPV), may increase the risk of developing atheromatous lesions on coronary arteries. However, there is a lack of data regarding the possible association between HPV infection and coronary artery disease (CAD) in women.

**Objective:**

To investigate whether HPV infection is associated with the occurrence of CAD among climacteric women.

**Methods:**

The presence of CAD and cervical HPV DNA was investigated in 52 climacteric women. Social and demographic variables and metabolic profiles were also investigated.

**Results:**

Among 27 women with CAD, 16 were positive for HPV, whereas 11 were negative. The presence of cervical HPV was strongly associated with CAD, after adjusting for demographic variables, health and sexual behaviors, comorbidities, and known cardiovascular risk factors. HPV-positive women showed a greater likelihood of having CAD (odds ratio [OR] = 3.74; 95% confidence interval [CI]: 1.16 to 11.96) as compared with HPV-negative women, particularly those infected with high-risk HPV types (OR = 4.90; 95% CI: 1.26 to 19.08).

**Conclusion:**

These results support the hypothesis that HPV infection might be associated with CAD among climacteric women, though further studies are needed to investigate the mechanisms involved.

## 1. Introduction

Cardiovascular diseases are the leading cause of death and constitute a major public health issue worldwide. Globally, they account for millions of deaths and, in Brazil, represent 29.4% of all deaths [1, 2]. Indeed, coronary artery disease (CAD) is characterized by insufficient blood supply to the heart through the coronary arteries, leading to myocardial infarction [3, 4]. Important risk factors for CAD are well established, including hypertension, diabetes, dyslipidemia, and smoking [2, 3, 5]. However, several individuals with CAD display none of these risk factors, thereby instigating the investigation of other determinants or associated variables [4, 5].

Infections with several agents such as HIV, Epstein-Barr virus, cytomegalovirus, and* Chlamydia pneumonia* have been suggested as risk factors for CAD, possibly by promoting a chronic inflammatory status [6, 7]. In this context, the human papillomavirus (HPV), one of the most common sexually transmitted infections worldwide, may play a significant role. HPV includes more than 200 subtypes, which are etiologically linked to the development of benign and malignant lesions. HPV 16 and 18 are the most common “high-risk” types and are strongly associated with cervical cancer and other malignancies [8]. In the general female population, the prevalence of HPV infection ranges 2-44% [8]. In developing countries, the prevalence ranges 21-48%, with high-risk HPV being detected in 48-53% of infected individuals [9–11]. Over the years, our group has specialized in HPV-related tumors, such as penile [12], anal [13], and cervical [14] cancers.

Previous reports [15] have described that women with vaginal HPV show a threefold increased risk of cardiovascular disease as compared with HPV-negative women. More recently, HPV DNA and proteins have been detected in 50% of atheromatous coronary arteries in a small sample of 20 deceased donors [16]. HPV infection is speculated to facilitate the development of CAD by providing an enhanced systemic inflammatory stimulus [16], which is also observed in HPV-transgenic animal models [17]. In fact, chronic unresolved inflammation plays a key role in CAD [18] and HPV infection might also contribute to this. Occasional studies claim that HPV may be found in circulating blood leukocytes [19] and endothelial cells [20], which might help locating the virus to atheromatous plaques. Another line of thought points out that HPV, like other viruses, interferes with lipid metabolism, which might also contribute to CAD [16, 21]. Therefore, the present study was aimed at investigating a possible association between HPV infection and CAD in climacteric women.

## 2. Methods

### 2.1. Ethics and Study Population

A cross-sectional study was approved by the Federal University of Maranhão Ethics Committee (approval #195.357). Written informed consent was obtained from all women. The sample size was calculated considering a prevalence of 65% of women with coronary artery disease positive for HPV [15], a power of 80% and a 5% significance level.

### 2.2. Inclusion and Exclusion Criteria

Volunteers with and without CAD were included if they were over 35 years of age and climacteric. Individuals with cardiac catheterization following coronary angioplasty were considered for enrolment and referred to the Gynecology Service of the Clinical Research Center/UFMA, where they underwent clinical examination to confirm eligibility. Exclusion criteria were previous chemotherapy/ pelvic radiotherapy or having serum HIV positivity.

### 2.3. Sociodemographic Variables

Sociodemographic variables (age, ethnicity, education, family income, professional activity, marital status, sexual behavior, alcohol consumption, smoking, and physical activity) were investigated using a semistructured questionnaire specifically developed for this study.

### 2.4. Comorbidities

During clinical examination, volunteers reported the presence or absence of comorbidities (hypertension, diabetes, thyroid disorders, and sexually transmitted diseases). Next, blood pressure (BP) and body mass index (BMI) were determined for each participant. For BP, volunteers were required to have an empty bladder and have not exercised or consumed alcohol, coffee or any food for at least 30 minutes prior to measurement. Systolic and diastolic BP were measured following the Brazilian Society of Cardiology guidelines, using a stethoscope and a mercury sphygmomanometer [22]. BP was considered abnormal when BP was equal to or greater than 130/85 mmHg. The BMI was calculated as the ratio between body weight in kilograms divided by the square of height in meters, and was categorized according to the WHO cut off points [23]: <18.5 (underweight), 18.5 to 24.9 (normal weight), 25.0 to 29.9 (overweight), and >30.0 (obese).

### 2.5. Metabolic Profile

Venous blood samples were obtained from volunteers after a 12-hour fasting period. After collection into tubes with and without anticoagulant, plasma and serum were separated by centrifugation and stored in freezer -80°C until further analysis. The samples were used to perform hemograms, glucose profile (fasting glucose, insulin, and glycated hemoglobin), lipid profile (total cholesterol, triglycerides, High Density Lipoprotein-HDL and Low Density Lipoprotein-LDL cholesterol), renal profile (creatinine and urea), and high-sensitivity C-reactive protein (hs-CRP).

### 2.6. HPV Detection and Genotyping

Cervical material was collected and submitted to nested polymerase chain reaction (PCR) for HPV genotyping. Extraction of genetic material was performed using the QIAamp DNA Mini and Blood Mini kit (QIAGEN, Valencia, CA) following the manufacturer's instructions. Briefly, 400 *μ*L sample solution was added to 400 *μ*L of AL buffer and 20 *μ*L of proteinase. Then, 400*μ*l of 100% ethanol was added to each tube; the samples were centrifuged four times and, after a brief incubation period, were again centrifuged at 8,000 rpm for 5 minutes to elute the DNA, which was stored at -20°C. The DNA concentration was determined using a NanoVue (GE) spectrophotometer. HPV DNA detection was performed by* L1* gene amplification using a PCR-NESTED technique and the primer pairs PGMY 09/11 and GP 5 + / 6 +. Next, the product of the first reaction with the primers GP5 + / GP6 + was amplified.

Following the PCR reactions, 5 *μ*L of each sample was submitted to 1.5% agarose gel electrophoresis to verify amplification. The PCR products were purified and subsequently sequenced using a MegaBACE 1000 automated sequencer (GE Healthcare, UK). Chromatograms of the sequences obtained were analyzed by Chromas software (Technelysium) and the sequences were submitted to online BLASTn software for the identification of HPV types. A total of 36 DNA-HPV samples were analyzed, and HPV types 16, 18, 33, 35, 39, 45, 51, 56, and 58 were defined as posing high oncogenic risk.

### 2.7. Statistical Analysis

Data are presented as absolute and relative frequencies. A binary logistic regression model was used to obtain adjusted odds ratio (OR) and confidence intervals (CI). Data were analyzed using EpIInfo 7 software (CDC, Atlanta). A *p* < 0.05 was considered statistically significant.

## 3. Results

Fifty-two women participated in this study, who were divided into two groups, according to the presence or absence of HPV infection: HPV-positive (n = 23) and HPV-negative (n = 29) (Table 1). The profile of HPV-positive volunteers included an age range of 50 to 59 years, single, low socioeconomic status, no drinking or smoking history, sedentary, being with early beginning of sexual activity (12-18 years), having few sexual partners (one), and being without sexual activity. There were no differences between the HPV-positive and HPV-negative groups with regard to sociodemographic variables.

Variables related to metabolic risk for developing CAD in the both groups are summarized in Table 2. There was no statistical difference between the two groups regarding the prevalence of diabetes mellitus or metabolic syndrome and values of hs-CRP, body mass index, blood glucose, and triglycerides. However, the HPV-positive group showed lower blood levels of HDL cholesterol (*p *= 0.008) and elevated systemic blood pressure (*p *= 0.002) when compared with the HPV-negative group.

Regarding the presence of HPV, 44.2% of all women were positive for cervical HPV DNA, 69.6% of which with CAD, and 30.4 % without CAD (Table 2). Among HPV-negative women, the CAD prevalence was 37.9%. The difference in CAD prevalence between the HPV-positive and negative groups was marginally significant (*p *= 0.0467) and the OR for CAD was 3.74 for HPV-positive volunteers compared with HPV-negative women.

Sixteen women (30.8%) had high-risk HPV types (16, 18, 33, 39, 45, and 58). These volunteers included 44.5% of the women with CAD and 16.0% of those without CAD (Table 3). After adjusting for age and ethnicity, the presence of high-risk vaginal HPV DNA was statistically associated with CAD (OR = 4.90,* p* = 0.0384). The presence of other types of HPV was not associated with CAD in this study.

## 4. Discussion

HPV infection is the cause of multiple benign and malignant lesions, but a possible association with cardiovascular disease has seldom been proposed and remains speculative. Still, some evidence [15] has reported that vaginal HPV infections increase the risk of cardiovascular diseases. One study [16] identified HPV in 55% of atheromatous coronary arteries in a small sample of postmortem donors, and the HPV E7 protein was detected in smooth muscle cells, plasma cells, and foamy macrophages located in those plaques. These observations raise questions concerning the possible mechanisms by which HPV could promote the formation of atheromatous plaques.

The prevailing view is that HPV remains confined to its epithelial lesions, but some authors report the detection of HPV DNA and proteins on endothelial cells [20] and circulating blood leukocytes [19]. If these observations reflect true viral dissemination, they could help explaining the presence of HPV in atheromatous lesions [16]. On the other hand, if HPV remains confined to mucosal lesions, it may be involved in CAD by promoting a systemic inflammatory status [17] and by deregulating the host metabolism, in particular that of lipids [21]. In the present study, we chose to address some of these issues in climacteric women. Menopause is associated with chronic inflammation and has major implications for CAD development [24, 25] and associated lipid disorders [26]. HPV incidence is also higher in this group [27] and might act synergistically with some of these factors to aggravate its cardiovascular risk.

Among climacteric women, we observed a marginally significant association between HPV infection and CAD. Importantly, the high-risk HPV genotypes associated with cancer were selectively associated with CAD, whereas no association with low-risk genotypes was observed. This is in line with previous studies [16] that detected the two most common high-risk HPV types, HPV16 and HPV18, in atheromatous plaques, but not low-risk types. Interestingly, the HPV type observed in endothelial cells and blood leukocytes in previous studies [19, 20] was also the high-risk HPV16.

The present results support the hypothesis that infection with HPV is associated with CAD, and that there is a specific association with high-risk (rather than low-risk) HPV types. If HPV does reach coronary arteries and is present in atheromatous plaques, it might promote their development through its classical cellular targets, p53, and the retinoblastoma protein (pRb). The loss of p53 function in macrophages was found to be strongly associated with the increase of atherosclerotic lesions [28–30]. In addition, knock-out animals for pRb showed increased development of atherosclerosis [31].

The present results also show a lower HDL and higher systemic blood pressure levels in HPV-positive compared with the HPV-negative group. While this could represent a menopause-related change, the statistical correlations with HPV infection were highly significant and call for an explanation, which may involve the interference of HPV on lipid metabolism [21]. However, the reduced number of subjects in our study and the restriction to climacteric women only and the lack of further cardiovascular data do not allow us to draw conclusions in this regard.

## 5. Conclusions

Overall, in the current study, HPV infection was associated with a greater likelihood of CAD occurrence among climacteric women. Prospective cohort studies are hereby necessary to investigate a potential causal effect of HPV infection on CAD development, and the investigation of biological mechanisms underlying this association is warranted.

## Figures and Tables

**Table 1:** Association between sociodemographic characteristics of participants and HPV status.

Variable	HPV positive (n=23)	HPV negative (n=29)	*p*	OR (95% CI)
*Age group*				
37–49	6 (26.1)	4 (13.8)	0.4454	Ref
50–59	9 (39.1)	19 (65.5)		0.31 (0.07-1.40)
60+	8 (34.8)	6 (20.7)		0.88 (0.17-4.62)
*Marital Status*				
Single	12 (52.2)	13 (44.8)	0.9336	Ref
Married	7 (30.4)	15 (51.7)		0.55 (0.16-1.85)
Widow	4 (17.4)	1 (3.5)		4.72 (0.45-8.77)
*Race*				
White	9 (39.1)	12 (41.4)	0.3268	Ref
Brown	10 (43.5)	13 (44.8)		1 (0.43-2.30)
Black	4 (17.4)	4 (13.8)		0.36 (0.11-1.14)
*Income*				
1 MW	12 (52.2)	11 (37.9)	0.1686	1.16 (0.43-2.30)
2-3 MW	5 (21.7)	11 (37.9)		1 (0.32-3.10)
4+ MW	6 (26.1)	7 (24.2)		Ref
*Alcoholism*				
Yes	4 (17.4)	2 (6.9)	0.3447	0.17 (0.05-0.60)
No	11 (47.8)	20 (69.0)		Ref
Ex alcoholic	8 (34.8)	7 (24.1)		0.47 (0.20-1.09)
*Smoking*				
Yes	-	1 (3.5)	0.0022	-
No	17 (73.9)	24 (82.7)		Ref
Former smoker	6 (26.1)	4 (13.8)		0.15 (0.04-0.50)
*Physical activity*				
Yes	8 (34.8)	7 (24.1)	0.5937	Ref
No	15 (65.2)	22 (75.9)		1.67 (0.50-5.61)
*Beginning of sexual activity*				
12 – 18	11 (47.8)	18 (62.1)	0.5562	Ref
19 – 25	8 (34.8)	11 (37.9)		0.6 (0.26-1.37)
25 +	4 (17.4)	-		0.13 (0.33-0.58)
*Sexual partners*				
1	11 (47.8)	14 (48.3)	0.8445	Ref
2	6 (26.2)	8 (27.6)		0.46 (0.17-1.21)
3	3 (11.5)	2 (6.9)		0.23 (0.06-0.80)
4 +	3 (11.5)	5 (17.2)		0.30 (0.10-0.94)
*Actual sexual activity*				
Yes	7 (30.4)	14 (48.3)	0.3912	0.46 (0.14-1.47)
No	16 (69.6)	15 (51.7)		Ref

OR: odds ratio; CI: confidence interval; MW: minimum wage; Ref: reference.

**Table 2:** Association between clinical variables of participants and HPV status.

Variable	HPV positive (n=23)	HPV negative (n=29)	*p*	OR (95% CI)
*BMI, kg/m* ^*2*^				
< 30	14 (60.9)	21 (72.4)	0.2865	Ref
≥ 30	9 (39.1)	8 (27.6)		0.57 (0.23–1.36)
*Blood pressure, mmHg*				
< 130 x 85	4 (17.4)	11 (37.9)	0.002	Ref
≥ 130 x 85	19 (82.6)	18 (62.1)		4.5 (1.52 – 12.29)
*Triglycerides, mg/dL*				
< 150	13 (56.6)	19 (65.5)	0.6767	Ref
≥ 150	10 (43.4)	10 (34.5)		0.7692 (0.33–1.75)
*HDL Cholesterol, mg/dL*				
≥ 50	5 (21.7)	10 (34.5)	0.008	Ref
< 50	18 (78.3)	19 (65.5)		3.8 (1.41 – 10.37)
*Blood glucose, mg/dL*				
< 100	14 (60.9)	16 (55.2)	0.8473	Ref
≥ 100	9 (39.1)	13 (44.8)		0.92 (0.43 – 1.97)
*HB1AC, %*				
< 5,4	4 (17.4)	6 (20.7)	0.7665	Ref
≥ 5,4	19 (82.6)	23 (79.3)		1.23 (0.30-5.04)
*hs-CRP, mg/dL*				
< 0,3	9 (39.1)	15 (51.7)	0.4042	Ref
≥ 0,3	14 (60.9)	14 (48.3)		1.55 (0.67 – 3.59)
*Metabolic Syndrome*				
No	13 (56.6)	18 (62.1)	0.8383	Ref
Yes	10 (43.4)	11 (37.9)		0.84 (0.34 – 2.04)
*CAD*				
No	7 (30.4)	18 (62.1)	0.0467	Ref
Yes	16 (69.6)	11 (37.9)		3.74 (1.16-11.96)

OR: odds ratio; CI: confidence interval, BMI: body mass index; Ref: reference.

**Table 3:** Association between HPV infection and the occurrence of CAD.

Variable	With CAD (n=27)	Without CAD (n=25)	*p*	OR (95% CI)
*HPV *				
Negative	11 (40.7)	18 (72.0)		Ref
High-risk	12 (44.5)	4 (16.0)	0.0384	4.90 (1.26-19.08)
Other types	4 (14.8)	3 (12.0)	0.6182	2.18 (0.40-11.64)

OR: odds ratio; CI: confidence interval; Ref: reference.

## Data Availability

The data used to support the findings of this study are available from the corresponding author upon request.
